# Sodium Polyoxotungstate Inhibits the Replication of Influenza Virus by Blocking the Nuclear Import of vRNP

**DOI:** 10.3390/microorganisms12051017

**Published:** 2024-05-17

**Authors:** Zhuogang Li, Yuanyuan Duan, Yang Yu, Yue Su, Mingxin Zhang, Yarou Gao, Lefang Jiang, Haonan Zhang, Xiaoqin Lian, Xingjian Zhu, Jiaxin Ke, Qun Peng, Xulin Chen

**Affiliations:** Institute of Medical Microbiology, Department of Immunology and Microbiology, College of Life Science and Technology, Jinan University, Guangzhou 510632, China; qq1205720100@126.com (Z.L.); yyduan@jnu.edu.cn (Y.D.); yuyang_0821@163.com (Y.Y.); 15625583649@163.com (Y.S.); mxzhang@jnu.edu.cn (M.Z.); gyr960202@163.com (Y.G.); lfjiang28@163.com (L.J.); honan01@163.com (H.Z.); lxq536200@163.com (X.L.); 13349798871@163.com (X.Z.); 18259408216@163.com (J.K.); pengq027@jnu.edu.cn (Q.P.)

**Keywords:** sodium metatungstate, influenza virus, antiviral, nuclear import of vRNP, virion aggregation

## Abstract

Both pandemic and seasonal influenza are major health concerns, causing significant mortality and morbidity. Current influenza drugs primarily target viral neuraminidase and RNA polymerase, which are prone to drug resistance. Polyoxometalates (POMs) are metal cation clusters bridged by oxide anions. They have exhibited potent anti-tumor, antiviral, and antibacterial effects. They have remarkable activity against various DNA and RNA viruses, including human immunodeficiency virus, herpes simplex virus, hepatitis B and C viruses, dengue virus, and influenza virus. In this study, we have identified sodium polyoxotungstate (POM-1) from an ion channel inhibitor library. In vitro, POM-1 has been demonstrated to have potent antiviral activity against H1N1, H3N2, and oseltamivir-resistant H1N1 strains. POM-1 can cause virion aggregation during adsorption, as well as endocytosis. However, the aggregation is reversible; it does not interfere with virus adsorption and endocytosis. Our results suggest that POM-1 exerts its antiviral activity by inhibiting the nuclear import of viral ribonucleoprotein (vRNP). This distinct mechanism of action, combined with its wide range of efficacy, positions POM-1 as a promising therapeutic candidate for influenza treatment and warrants further investigation.

## 1. Introduction

Influenza virus infection poses a significant global health threat, leading to severe illness, hospitalizations, and even mortality, particularly among vulnerable populations such as the elderly, young children, and individuals with underlying health conditions [1,2,3,4,5]. The continuous evolution of influenza viruses through antigenic drift and shift presents challenges in the development of effective antiviral therapies [6,7]. Antiviral medications play a crucial role in managing and controlling influenza infections, especially in high-risk populations. Current antiviral options for influenza include neuraminidase inhibitors (oseltamivir, zanamivir, peramivir) and cap-dependent endonuclease inhibitors (baloxavir) [8]. However, the emergence of drug-resistant strains and the limited efficacy of existing antivirals underscore the necessity for novel antiviral agents with distinct mechanisms of action [9].

Polyoxometalates (POMs) are a diverse and vast family of polynuclear oxobridged early-transition metal compounds of (MO_x_) polyhedrals. M is generally W, Mo, V, Nb, or Ta in its highest oxidation number. There are two main families of POMs: the isopolyoxometalates [M_x_O_y_]^n−^ and the heteropolyoxometalates [X_x_M_y_O_z_]^n−^ (X is a heteroatom such as B, Si, or P). The connection of polyhedrals in different manners leads to various three-dimensional structures of POMs, such as Lindqvist ([M_6_O_19_]^n−^), Anderson–Evans ([XM_6_O_24_]^n−^), Keggin ([XM_12_O_40_]^n−^), Wells–Dawson ([X_2_M_18_O_62_]^n−^) or their derivatives [10,11]. POMs have exhibited various biological activities, including potent anti-tumor, antiviral, and antibacterial effects [12,13,14]. Moreover, POMs have demonstrated promising therapeutic potential for diseases such as diabetes and Alzheimer’s disease [15,16]. In the field of antiviral research, POMs have displayed remarkable activity against various DNA and RNA viruses [17,18], encompassing influenza virus [19], hepatitis B virus [20], hepatitis C virus (HCV) [21], herpes simplex virus (HSV) [22], dengue virus [23], and human immunodeficiency virus (HIV) [24].

Several POMs have been discovered to interact with host factors or viral proteins, demonstrating antiviral effects at various stages of viral replication. For example, PT-1 can obstruct the binding site of gp120 in the CD4 receptor or directly bind to gp41 NHR, thereby disrupting the HIV entry process [25]. Furthermore, as reported by Qi et al., POM-12 has the ability to impair HCV’s integrity by damaging the lipid layer of its viral envelope [21]. PM-19 can block the interaction between the HSV envelope protein (gD, glycosylated ectodomain) and cell surface membrane proteins to inhibit HSV infections [26]. PM-523 has also been identified as a potent inhibition of influenza A virus (IAV) replication in MDCK cells by inhibiting the fusion of the viral envelope and cell membrane [27].

Various POMs have unique structures and exhibit different drug activities. This study discovered sodium polyoxotungstate (POM-1) as an inhibitor against influenza virus infection from an ion channel inhibitor library. POM-1 has previously demonstrated anti-HIV activity [28]; however, its effectiveness against the influenza virus has not been reported. Here, the in vitro experiments have demonstrated the potent antiviral activity of POM-1 against H1N1, H3N2, and oseltamivir-resistant strains. Our mechanistic investigation suggests that POM-1 induces virion aggregation during adsorption and after endocytosis. Furthermore, POM-1 exerts its antiviral effect through a unique mechanism by inhibiting the nuclear import of viral ribonucleoprotein (vRNP). The distinctive mode of action combined with its broad efficacy profile positions POM-1 as a promising therapeutic candidate for influenza treatment, warranting further exploration.

## 2. Materials and Methods

### 2.1. Cell Lines and Virus Strains

The Madin–Darby canine kidney (MDCK) cells (ATCC CCL-34) and the human pulmonary epithelial (A549) cells (ATCC CCL-185) were maintained in Dulbecco’s modified Eagle’s medium (DMEM, Gibco, Grand Island, NY, USA) supplemented with 10% FBS (Meilunbio, Dalian, China), 1% penicillin/streptomycin (P/S) (Gibco, Rockville, MD, USA). All cells were maintained in a 5% CO_2_ incubator at 37 °C during passage and virus infection. Influenza virus strain A/Puerto Rico/8/1934 H1N1 virus (PR8), oseltamivir-resistant PR8 (H274Y oseltamivir-resistant), and H3N2 (A/human/Hubei/3/2005) were provided by the National Virus Resource Center (Wuhan, China). All experiments in this study were conducted in a biosafety-level-2 laboratory.

### 2.2. Chemicals

The sodium polyoxotungstate (POM-1) was purchased from Macklin (Shanghai, China). Stock solutions were prepared in deionized water (80% *w*/*v*) and stored at −40 °C. Oseltamivir acid was purchased from Toronto Research Chemicals (Toronto, ON, Canada). Stock solutions were prepared in DMSO and stored at −40 °C. For all compounds, the working concentrations were prepared by diluting stock solutions in a culture medium or reaction buffer immediately before use.

### 2.3. Antibodies

Mouse anti-nucleoprotein (NP) was purchased from Sino Biological (11675-MM03T, Beijing, China). Mouse anti-glyceraldehyde-3-phosphate dehydrogenase (GAPDH) (AF0006) and horseradish peroxidase (HRP)-conjugated anti-mouse IgG (A0216) were purchased from Beyotime Biotechnology (Shanghai, China). Alexa Fluor 488-conjugated goat anti-mouse secondary antibody was purchased from Cell Signaling Technology (4408S, Danvers, MA, USA). DAPI solution was purchased from Solarbio (C0060, Beijing, China).

### 2.4. Cell Viability Assay

MDCK or A549 cells were seeded in 96-well plates at a density of 1.5 × 10^4^ cells/well and cultured at 37 °C overnight. Then, the cells were treated with serially diluted POM-1 and incubated at 37 °C for 48 h. The cell viabilities were measured by a Cell Titer-Glo luminescent cell viability assay (Promega, Madison, WI, USA) based on quantification of the ATP, according to the manufacturer’s protocol. Luminescence signals were detected using a Varioskan LUX multimode microplate reader (Thermo, Waltham, MA, USA), and cell viability was normalized to the vehicle-treated cell. Experiments were performed three times for each condition. The CC_50_ for the compound was calculated from the resulting dose–response curves using GraphPad Prism 7.04.

### 2.5. Antiviral Assay

MDCK or A549 cells were seeded in 96-well plates at a density of 1.5 × 10^4^ cells/well and cultured overnight. The supernatant medium was discarded, and the cells were washed with PBS. In the presence of serially diluted POM-1, MDCK cells were infected with PR8 at a multiplicity of infection (MOI) of 0.02, and A549 cells were infected with PR8 at an MOI of 0.1. After incubation at 37 °C for 48 h, the inhibition of viral replication was measured by the modified neuraminidase activity (NA) assay [29]. The fluorescence intensity of supernatants harvested at 48 h post-infection (hpi) was measured with a Varioskan LUX multimode microplate reader (Thermo, Waltham, MA, USA) and the inhibition rate was expressed as the 50% effective inhibitory concentrations (EC_50_). For detection of infectious virus yield reduction, the supernatants were titrated through the TCID_50_ assay on MDCK cells, and the virus titers were calculated according to the method of Reed–Muench.

### 2.6. Indirect Immunofluorescence Assay (IFA) 

Treated cells on 96-well plates or glass-bottom dishes were washed with PBS and fixed with 4% paraformaldehyde (PFA) for 20 min at room temperature (RT). The fixed samples were incubated in PBS with 0.3% Triton X-100 (BioFroxx, Guangzhou, China) for 20 min at RT for permeabilization. Then, cells were blocked with 5% bovine serum albumin in PBS for 1 h at 37 °C. The cells were then incubated with a primary antibody against the influenza NP (1:1000) at 4 °C overnight. After three rinses, cells were incubated with Alexa Fluor 488-conjugated goat anti-mouse secondary antibody (1:1000) for 1 h in darkness at 37 °C, and cell nuclei were stained with DAPI (1:1000) for 10 min. After three final rinses with PBS, the samples on 96-well plates or glass-bottom dishes were examined using the Nikon Eclipse Ti2 inverted microscope (Nikon, Tokyo, Japan) or the Leica SP8 confocal microscope (Leica, Heerbrugg, Switzerland), respectively.

### 2.7. Western Blot

Cells on culture plates were harvested and lysed in radio-immunoprecipitation assay (RIPA) buffer. Lysates were centrifuged at 4 °C for 10 min at 12,000× *g*, and supernatants were collected. Protein concentrations were determined using a Pierce BCA protein assay kit (Biosharp, Hefei, China). Protein samples were then subjected to 12.5% sodium dodecyl sulfate–polyacrylamide gel electrophoresis (SDS-PAGE). The samples’ proteins were transferred onto polyvinylidene difluoride membranes. The membranes were blocked in PBS containing 5% (*w*/*v*) skim milk powder, followed by incubation with primary antibodies against the influenza NP (1:1000) and GAPDH (1:1000) at 4 °C for overnight. The membranes were washed three times with TBST and incubated with the HRP-conjugated secondary antibody (1:5000) at room temperature for 1 h. The target proteins were visualized using the Clarity Western ECL Substrate (Bio-Rad, Singapore). The signal was captured with a ChemiDoc™ imaging system (Bio-Rad, Singapore).

### 2.8. Time-of-Addition Assay

To investigate the stage of viral life cycle inhibited by POM-1, A549 cells were infected with PR8 virus at an MOI of 0.5 and incubated at 37 °C while adding POM-1 (50 μM) at different time points. The cells were fixed with 4% paraformaldehyde (PFA) at 10 h post-infection (hpi). Indirect immunofluorescence assay (IFA) was performed to detect NP expression and determine the inhibition of viral replication.

In a defined time-of-addition assay, A549 cells were precooled at 4 °C for 30 min before infection with PR8 (MOI = 1) at 4 °C for 1 h. For treatment during viral attachment, POM-1 (100 µM) was added during infection at 4 °C and discarded after infection. For other treatments, cells were incubated at 37 °C after infection at 4 °C, and POM-1 was added at different times. The cells were collected and lysed in RIPA buffer at 8 hpi. The NP expression was detected by Western blot to determine the inhibition of viral replication.

### 2.9. Virucidal Assay

For the virucidal experiment, the PR8 virus stock (10^6.5^ TCID_50_/mL) and medium containing POM-1 were mixed and incubated at 37 °C for 1 h. Then, the virus–compound mixtures were diluted 100-fold and used to infect MDCK cells. For controls, the virus and medium-containing compounds were separately incubated. Then, they were diluted 100-fold and mixed before infecting cells. The mixtures were titrated through the TCID_50_ assay, and the virus titers were calculated according to Reed–Muench’s method.

### 2.10. Virus Attachment Assay

To determine whether POM-1 affected the adsorption of the influenza virus on cells, A549 cells were seeded on glass-bottom dishes (NEST, Wuxi, China) overnight. The cells and solutions used were precooled at 4 °C for 30 min. Then, the cells were infected with PR8 either in the presence or absence of the compound at 4 °C for 1h. The inoculum was removed, and cells were washed with cold PBS and fixed with 4% PFA. Cells were stained for influenza NP and nuclei. Fluorescent images were acquired by confocal microscope.

For scanning electron microscopy (SEM) observation, cells were cultured on coverslips and fixed with precooled 2.5% glutaraldehyde solution (Coolaber, Beijing, China) for 2 h. After PBS rinses, cells were dehydrated in gradient ethanol series (10%, 30%, 50%, 70%, 90%, 100%, 100%; 10 min for each grade) and air-dried overnight at RT. The coverslips were mounted on an aluminum stub and coated with a gold film using a sputter coater. Then, samples were examined under the Zeiss ULTRA 55 field emission scanning electron microscope (Zeiss, Oberkochen, Germany).

### 2.11. Neuraminidase Inhibition Assay

2′-(4-Methylumbelliferyl)-α-D-N-acetylneuraminic acid (MUNANA), a fluorescent substrate for the neuraminidase (NA), was used to determine the inhibition effect of POM-1 on NA activity of influenza viruses. PR8 virus was added to a 96-well culture plate and then mixed with serial diluted compound (in PBS) at 37 °C for 1 h. Oseltamivir was used as the positive control. Then, the virus–compound mixtures were transferred to a black opaque 96-well plate and mixed with 40 μM of MUNANA dissolved in MES solution (33 mM 2-[N-morpholino] ethanesulfonic acid and 4 mM CaCl_2_, pH = 6.5) at a volume of 40 μL:20 μL for each well, followed by incubation at 37 °C for 1 h. Then, 60 μL of stop solution (0.14 M NaOH in 83% ethanol) was added to each well to terminate the reaction. Fluorescence intensity was measured at an excitation wavelength of 370 nm and an emission wavelength of 450 nm under the Varioskan LUX multimode microplate reader (Thermo fisher, Singapore).

### 2.12. Virus Release Assay

A549 cells were seeded in 12-well plates at a 1.7 × 10^5^ cells/well density and cultured overnight. Cells were infected with PR8 at an MOI 0.5 and culture for 6 h. The supernatant medium was discarded, and the cells were washed with PBS. The fresh DMEM medium containing POM-1 was added, and cells were cultured for another 4 h. Then, supernatants were harvested, and the compound contained in supernatants was removed by dialysis. The solution selected was PBS, and dialysis was carried out three times at 4 °C for durations of 8 h, 8 h, and 13 h, respectively. After dialysis, the supernatants were titrated through the TCID_50_ assay, and the virus titers were calculated according to Reed–Muench’s method. The supernatants without dialysis treatment served as the control group.

To detect the virions in supernatants by Western blotting, the supernatant and precooled trichloroacetic acid solution (60% *w*/*v*) were mixed at a ratio of 2:1 to precipitate the protein. The mixtures were placed on ice for 30 min, then centrifuged at 20,000× *g* for 30 min at 4 °C. The supernatant was slowly decanted, followed by two washes with precooled acetone. Specifically, after adding acetone, the mixtures were placed on ice for 10 min, centrifuged at 20,000× *g* for 15 min at 4 °C, the acetone was decanted, and the pellet was air-dried at room temperature. Subsequently, the pellet was resuspended to prepare the total protein sample from the supernatant. The samples were then subjected to Western blotting for the detection of influenza NP.

### 2.13. Statistical Analyses

Data are presented as mean ± SEM. For all analyses, multiple independent experiments (N = 3) were carried out. The dose–response curves were obtained using nonlinear regression, log [drug] vs. response, and variable slope (four parameters) in GraphPad Prism 7.04. The CC_50_ (50% cytotoxic concentrations), EC_50_ (50% effective concentrations), and IC_50_ (50% inhibitory concentration) for the compound were calculated from the resulting curves using the software. The selective index (SI) was calculated as the ratio of the CC_50_ to the EC_50_. Differences between experimental groups were statistically analyzed using one-way ANOVA tests or unpaired *t*-tests, with *p*-values < 0.05 being statistically significant. Symbols for *p*-values used in the figures: * *p* < 0.05, ** *p* < 0.01, *** *p* < 0.001, and **** *p* < 0.0001.

## 3. Results

### 3.1. POM-1 Inhibits the Replication of Influenza Viruses In Vitro

**Figure 1:**
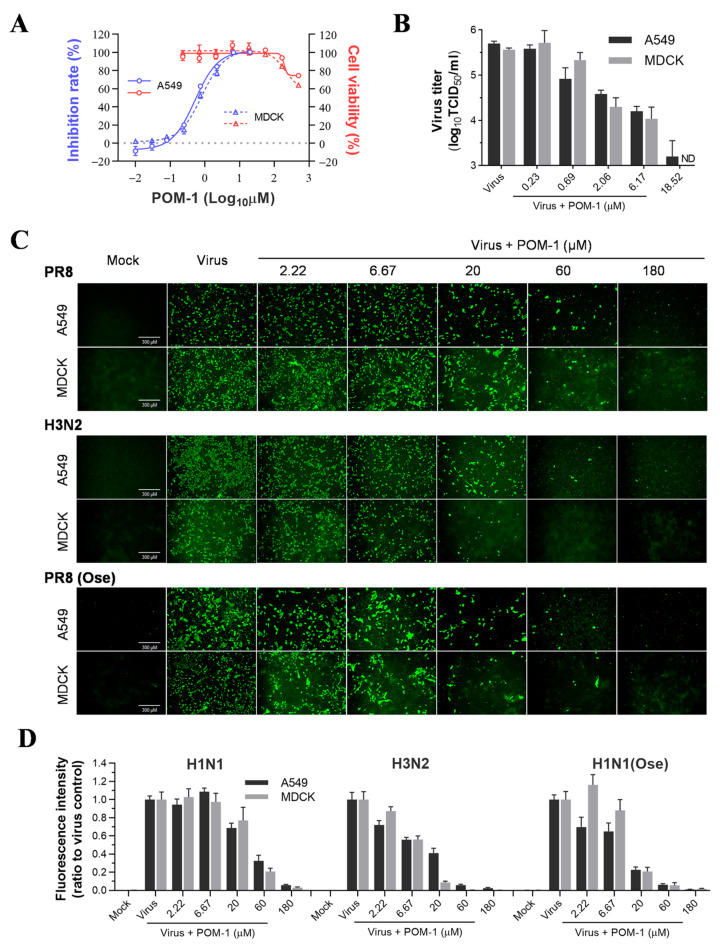
POM-1 inhibits influenza virus replication in vitro. (**A**) A549 and MDCK cells were infected with the PR8 virus at a multiplicity of infection (MOI) of 0.1 and 0.02, respectively, and serially diluted POM-1 was added during infection. After 48 h of incubation at 37 °C, supernatants were collected, the inhibitory effects of POM-1 on virus replication were determined based on the reduction in NA activities using NA activity assay. Cell viability was determined by Cell Titer-Glo assay after 48 h of exposure to POM-1 at the indicated concentrations. (**B**) The production of infectious virions in supernatants after treatment with serially diluted POM-1 was determined by TCID_50_ assay. ND is not detectable. (**C**) A549 and MDCK cells were infected with 0.1 or 0.02 MOI of H1N1, H3N2, or oseltamivir-resistant H1N1 virus (H1N1(Ose)) and treated with various concentrations of POM-1. At 24 hpi, the cells were fixed with 4% PFA, and the influenza virus NP expression was detected by IFA using a monoclonal NP antibody and imaged using a Nikon Eclipse Ti2 inverted microscope. (**D**) The fluorescence intensities were quantified using ImageJ software (version 7.04) and normalized to virus control. Values are expressed as the means ± SEM of three independent experiments.

We initiated the in vitro efficacy study of POM-1 by assessing its cytotoxicity on MDCK and A549 cells. No discernible cytotoxic effects were observed at concentrations of 55.56 µM and below for both cell lines. However, concentrations exceeding 166.67 µM resulted in significant cytotoxicity on MDCK and A549 cells. The CC_50_ values for these cell lines slightly exceeded 500 µM. To evaluate the antiviral activity of POM-1, we infected MDCK and A549 cells with PR8 virus and treated them with serially diluted POM-1. Subsequently, we measured the neuraminidase activity of the supernatant virions (Figure 1A). Our findings demonstrate that POM-1 effectively inhibits PR8 replication on MDCK and A549 cells, exhibiting EC_50_ values of 0.82 μM and 0.52 μM, respectively. Consequently, the selective indices for POM-1 on MDCK and A549 cells are calculated as 610 and 962, respectively, indicating potent inhibition of influenza virus replication by POM-1. 

To further validate the antiviral efficacy of POM-1, we evaluated its impact on the production of infectious virions in MDCK and A549 cells. As shown in Figure 1B, POM-1 demonstrated a dose-dependent inhibition of virus production, leading to a remarkable reduction exceeding 99% at a concentration of 18.52 µM in both cell lines. These findings indicate that the antiviral activity exerted by POM-1 is not influenced by cellular context.

Subsequently, we performed a study to compare the effectiveness of POM-1 in treating H3N2 and H1N1 influenza viruses, wild-type and oseltamivir-resistant strains, in A549 and MDCK cells. We used IFA to evaluate the expression of viral nucleoprotein (NP). Our findings showed that POM-1 has a broad-spectrum antiviral effect against different subtypes and oseltamivir-resistant influenza A viruses, as it effectively inhibited the expression of NP in both A549 and MDCK cells in a dose-dependent manner for all the virus strains tested (refer to Figure 1C,D). Collectively, our findings demonstrate the potent inhibitory effect of POM-1 on influenza virus replication in vitro.

### 3.2. POM-1 Inhibits Influenza Virus Replication in the Early Stage of Its Life Cycle

**Figure 2:**
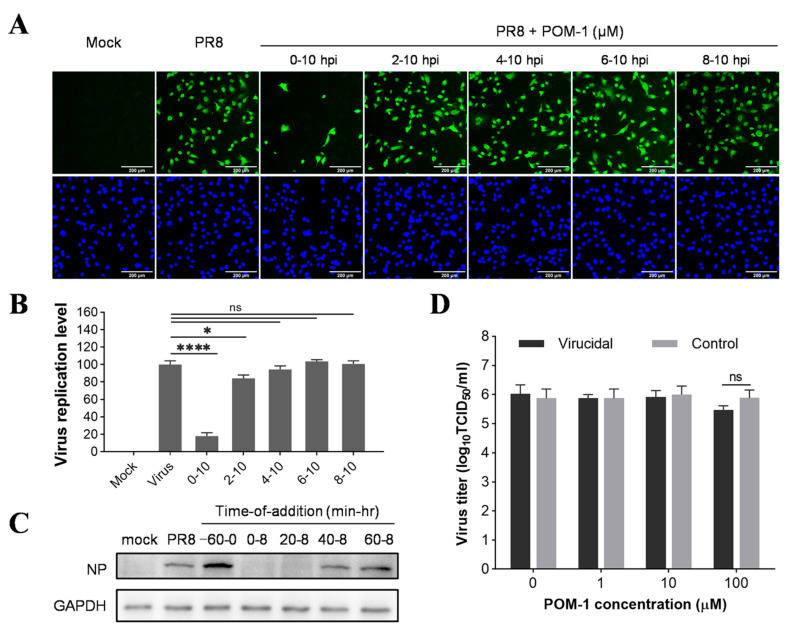
POM-1 inhibits influenza virus replication in the early stage of its life cycle. (**A**) A549 cells were infected with the PR8 virus at an MOI of 0.5, and 50 μM POM-1 was added at the indicated times. At 10 hpi, the cells were fixed and stained with anti-NP antibody and Alexa Fluor 488-labeled secondary antibody, followed by 4′,6-diamidino-2-phenylindole (DAPI) staining. The virus replication level, represented by the expression of virus NP, was determined by IFA and visualized under a Nikon Eclipse Ti2 inverted microscope. (**B**) The virus replication level was quantified by calculating the ratios of infected cells per field and normalizing them to virus control. Values are expressed as the means ± SEM of three independent experiments. The statistical significance of the results is indicated by * *p* < 0.05, **** *p* < 0.0001. ns, not significant when *p* > 0.05. (**C**) A549 cells were precooled at 4 °C for 30 min before infection with PR8 (MOI = 1) at 4 °C for 1 h. For pretreatment of POM-1 during viral attachment (−60–0 min) at 4 °C, and treatment of POM-1 (100 µM) during which POM-1 was added at different times post-virus infection, the whole-cell lysates were collected at 8 hpi, and the viral NP protein levels were determined by Western blotting. (**D**) In the virucidal assay, the PR8 virus solutions were incubated with different concentrations of POM-1 at 37 °C for 1 h, followed by being diluted and titrated using the TCID_50_ assay. For controls, the virus- and medium-containing compounds were separately incubated at 37 °C for 1 h. Then, they were diluted and mixed before titration. Values are expressed as the means ± SEM of three independent experiments.

To determine whether the antiviral effect of POM-1 is virucidal, we incubated influenza viruses with varying concentrations of POM-1 at 37 °C for 1 h and subsequently determined the viral titers using the TCID_50_ assay. Our results revealed that even at concentrations as high as 100 μM, POM-1 did not exert any discernible impact on virus titers (Figure 2D), indicating its inability to directly inhibit influenza virus replication by inactivating the virions.

To investigate the antiviral mechanism of POM-1, we conducted a time-of-addition assay to determine its impact on different stages of the influenza virus life cycle. A549 cells were infected with influenza virus at an MOI of 0.5 for one replication cycle, followed by incubation at 37 °C for 10 h. POM-1 was added to the culture medium at various times during this process. The inhibitory effect was assessed by evaluating the expression of NP protein using IFA. Compared to untreated cells, the addition of POM-1 at the onset of virus infection (0–10 hpi) resulted in almost complete inhibition of NP expression (Figure 2A). Conversely, when POM-1 was added at or after 2 hpi, minimal impact on NP expression was observed. Quantitative analysis of infected cells under different time-of-addition conditions further confirmed these findings, presented in Figure 2B. Additionally, Western blot analysis as part of a more precise time-of-addition assay corroborated these observations (Figure 2C), demonstrating that POM-1 exerts its antiviral activity during the early stages of the influenza virus life cycle.

### 3.3. POM-1 Does Not Block Virus Adsorption, Though It Triggers Virion Aggregation

**Figure 3:**
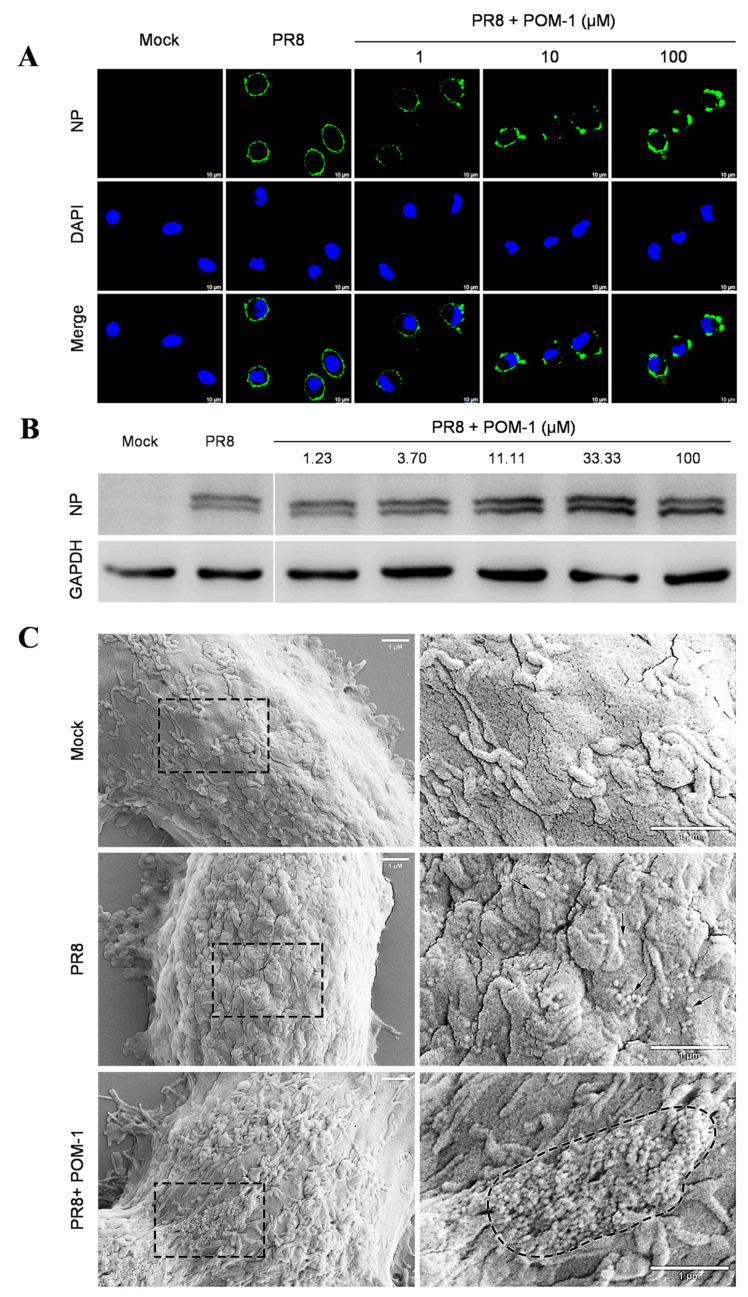

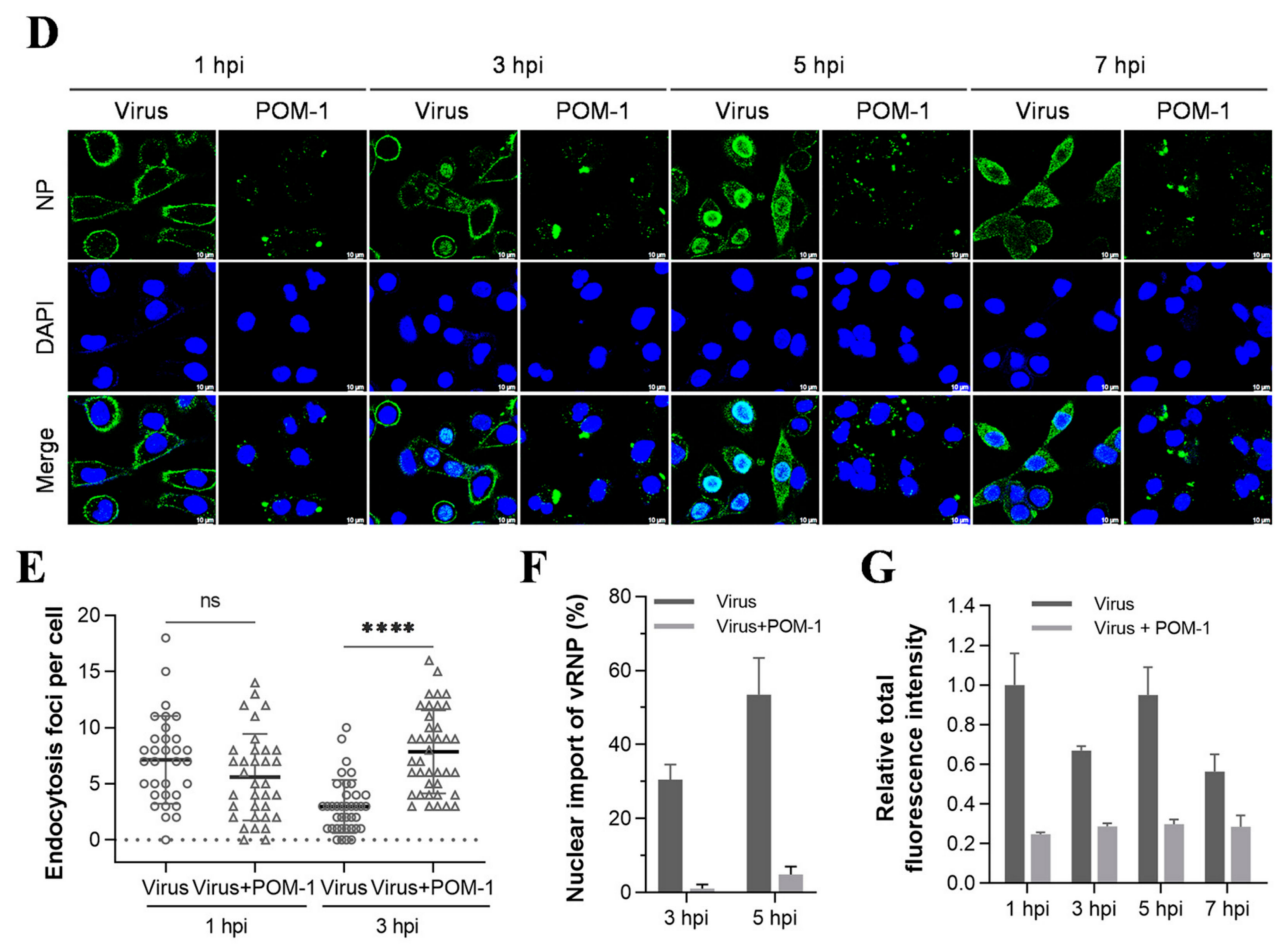
POM-1 causes virion aggregation during adsorption to host cells and blocks the nuclear import of vRNP. (**A**) A549 cells were precooled at 4 °C for 30 min, then infected with PR8 at an MOI of 10 in the absence or presence of POM-1 at indicated concentrations, and incubated at 4 °C for 1 h. The cells were fixed with 4% PFA, NP was fluorescently stained (green) using IFA, and the nucleus was DAPI-stained (blue) and imaged using a Leica SP8 confocal microscope. (**B**) After viral adsorption (MOI = 0.5) at 4 °C for 1 h in the absence or the presence of POM-1 at indicated concentrations, A549 cells were lysed immediately, and NP protein levels were determined by Western blotting. (**C**) The A549 cells were attached by PR8 virus (MOI = 2) for 1 h in the absence or presence of 100 µM POM-1 at 4 °C. Then, cells were fixed, dehydrated, coated, and imaged with a scanning electron microscope. The rectangular line-selected area is enlarged and displayed on the right, while arrows point out scattered virions, and the aggregated virions are indicated by an oval dotted line. (**D**) A549 cells were infected with PR8 at an MOI 1 and treated with 100 µM POM-1 at 37 °C until fixed at 1, 3, 5, and 7 hpi., respectively. Viral NP (green) and nuclei (blue) were visualized using confocal microscopy. (**E**) The endocytosis in (**D**) was determined by counting the number of NP fluorescent foci in the cytoplasm per cell. The numbers of counted cells at 1 hpi for the virus control group and POM-1-treated group were 35 and 34, respectively, and for 3 hpi, they were 38 and 39. (**F**) Infected cells containing NP nuclei were considered the import of vRNP, while the percentage of cells with infected nuclei in (**D**) was calculated. The number of cells counted for the virus control group and POM-1-treated group at 3 hpi were 95 and 91, respectively, while at 5 hpi, they were 73 and 106. (**G**) The total fluorescence intensities per field in (**D**) were quantified using ImageJ software and normalized to virus control at 1 hpi. Values are expressed as the means ± SEM of three independent experiments. The statistical significance of the results is indicated by **** *p* < 0.0001; ns, not significant.

The early stages of the influenza virus life cycle encompass multiple steps, including virus adsorption onto host cells followed by endocytosis, leading to acidification within vesicles that trigger membrane fusion for release into the cytoplasmic space as viral ribonucleoprotein complexes (vRNPs), ultimately culminating in their nuclear importation. To investigate the mechanism of action, we examined the impact of POM-1 on virus adsorption to A549 cells at 4 °C. Our findings revealed that untreated control cells displayed uniform binding patterns across their surfaces. In contrast, treatment with increasing concentrations of POM-1 resulted in progressively larger aggregates forming on cell membranes (Figure 3A). Notably, POM-1 did not diminish overall levels or kinetics associated with initial attachment events; instead, high concentrations of POM-1 slightly enhanced virus attachment, as confirmed by Western blot analyses (Figure 3B). These results indicate that POM-1 does not affect virus adsorption but concentration-dependently induces virion aggregation.

Next, we conducted a study to confirm our hypothesis that POM-1 can cause virion aggregation. We used scanning electron microscopy to observe influenza virions’ attachment to cells. In Figure 3C, we found POM-1-treated influenza virions attached to the cell surface as large aggregates. In contrast, untreated virions were attached as dispersed entities. These results provide strong evidence for the role of POM-1 in inducing virion aggregation. However, we found that virion aggregation does not affect virus adsorption.

### 3.4. POM-1 Does Not Affect Virus Endocytosis, While the Nuclear Import of vRNP Is Blocked

Next, we investigated whether POM-1 affects virus endocytosis and the nuclear import of vRNP by IFA of NP during one-cycle replication at 37 °C. We compared POM-1-treated virus-infected cells to those untreated at 1, 3, 5, and 7 hpi. The attached virions at 1 and 3 hpi appeared significantly decreased upon POM-1 treatment. Furthermore, at 3, 5, and 7 hpi, the nuclear import of vRNP was blocked (Figure 3D).

We subsequently performed a quantitative analysis to assess the impact of POM-1 on influenza virus endocytosis at 1 and 3 hpi (Figure 3E), nuclear import of vRNP at 3 and 5 hpi (Figure 3F), as well as the relative total fluorescence intensity of cells at 1, 3, 5, and 7 hpi (Figure 3G). Our findings reveal that treatment with POM-1 does not influence endocytosis at the early stage of infection (1 hpi). However, at later time points (3 hpi), we observed an augmented level of endocytosis in POM-1-treated cells compared to untreated ones. This enhanced endocytic activity may contribute to an increased uptake of aggregated virions under higher MOI conditions combined with POM-1 treatment. Furthermore, we noticed that part of the released vRNP entered the nuclei in untreated cells. Interestingly, POM-1 significantly impeded the nuclear importation of vRNP at 3 and 5 hpi. Finally, when comparing the total fluorescence intensity between treated and untreated cells at different time points (i.e., from 1 to 7 hpi), we found a significant reduction in NP levels in POM-1-treated cells (Figure 3G). These observations suggest that POM-1 impairs the nuclear importation of vRNP along with viral replication while potentially promoting degradation mechanisms for trapped virions.

Therefore, our results indicate that POM-1 does not affect virus endocytosis while blocking the nuclear import of influenza virus vRNP.

### 3.5. The Aggregation of Virions Caused by POM-1 during Adsorption Is Reversible

**Figure 4:**
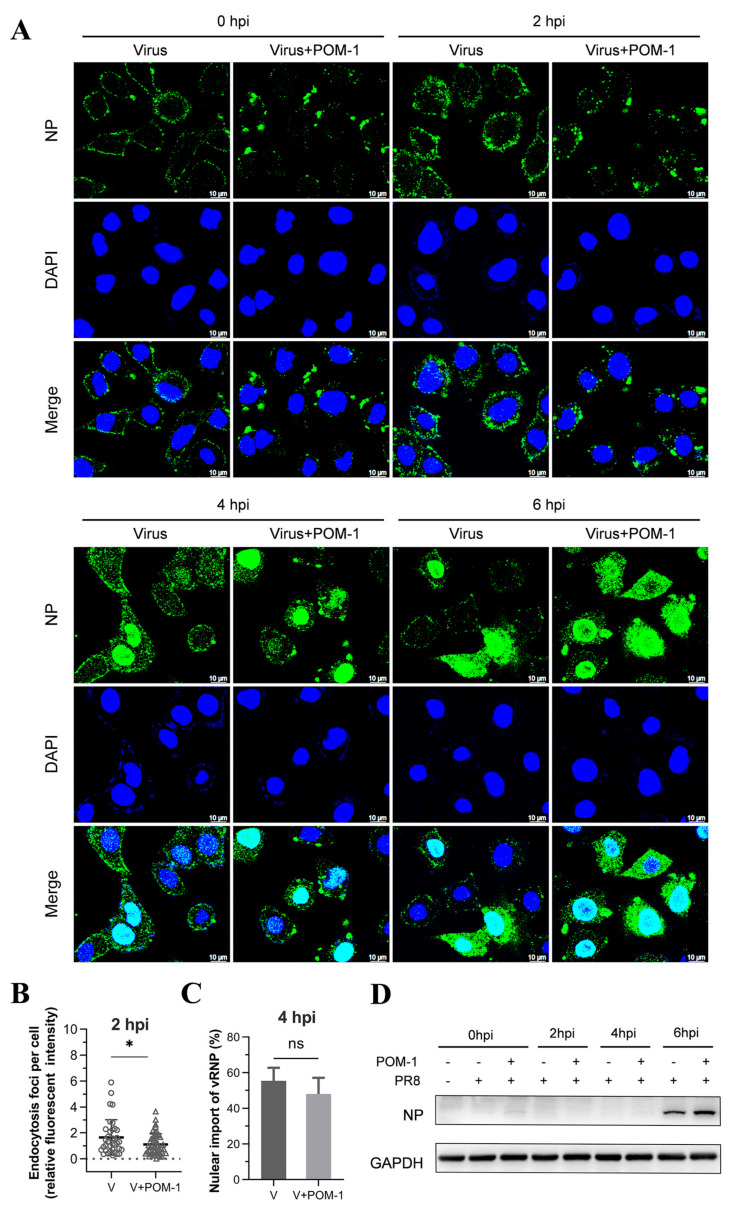
The aggregation of virions caused by POM-1 during adsorption is reversible if POM-1 is removed. (**A**) A549 cells were infected with PR8 and treated with 100 µM POM-1 at 4 °C for 1 h. After removing the supernatants, the cells were washed twice with PBS before being cultured in a fresh medium at 37 °C. Infected cells (MOI = 5) were fixed at different time points post-infection, and NP was fluorescently stained (green) using IFA, while the nucleus was stained (blue) with DAPI. Imaging was performed using a Leica SP8 confocal microscope. (**B**) The endocytosis in (**A**) was determined by quantifying the relative fluorescent intensities of NP foci in cytoplasm per cell. The number of counted cells at 2 hpi for virus control group and POM-1-treated group were 36 and 46, respectively. (**C**) Infected nuclei containing NP were considered imports of vRNP, while the percent of cells with infected nuclei in (**A**) were calculated per field. The number of counted cells at 4 hpi for the virus control group and POM-1-treated group were 79 and 79, respectively. Values are expressed as the means ± SEM of three independent experiments. The statistical significance of the results is indicated by * *p* < 0.05; ns, not significant. (**D**) A549 cells were treated as described in (**A**), except that the infected cells (MOI = 1) were lysed, and NP protein levels at different times post-infection were determined by Western blotting.

Subsequently, we performed a further virus replication assay to determine whether virion aggregation during adsorption is reversible. Influenza viruses were adsorbed to A549 cells in the presence of POM-1 at 4 °C, followed by PBS solution wash to eliminate unattached viruses. The cells were then cultured at 37 °C, and virus replication was assessed by examining NP expression and localization using IFA at different time points post-virus infection. As depicted in Figure 4A, treatment with POM-1 at 4 °C induced virion aggregation on the cell surface at 0 hpi; however, the removal of POM-1 gradually reduced the size of virion aggregates over time. The endocytosis at 2 and 4 hpi and the nuclear import vRNP at 4 and 6 hpi in POM-1-treated (during adsorption) cells were similar to that of the untreated cells.

We subsequently performed a quantitative analysis to assess whether virion aggregation during adsorption is reversible upon POM-1 removal based on the influenza virus endocytosis at 2 hpi (Figure 4B) and nuclear import of vRNA at 4 hpi (Figure 4C). We found that the influenza virus endocytosis and nuclear import of vRNA were comparable, suggesting that removing POM-1 after adsorption leads to reversible virion aggregation and restoration of vRNP nuclear import.

To validate the restoration of virus replication capacity upon POM-1 removal after adsorption, we conducted a virus replication experiment and assessed viral NP expression through Western blot analysis. The results demonstrated that the elimination of POM-1 after virus adsorption restored viral NP expression in infected cells to levels comparable with untreated cells, indicating unaffected upstream events such as endocytosis and nuclear import after POM-1 removal (Figure 4D). Our findings reveal that treatment with POM-1 does not influence endocytosis at the early stage of infection (1 hpi). Consequently, our findings establish the reversibility of virion aggregation during adsorption upon POM-1 elimination.

### 3.6. POM-1 Causes Virion Aggregation in the Cytoplasm and Hinders the Nuclear Import of vRNP

**Figure 5:**
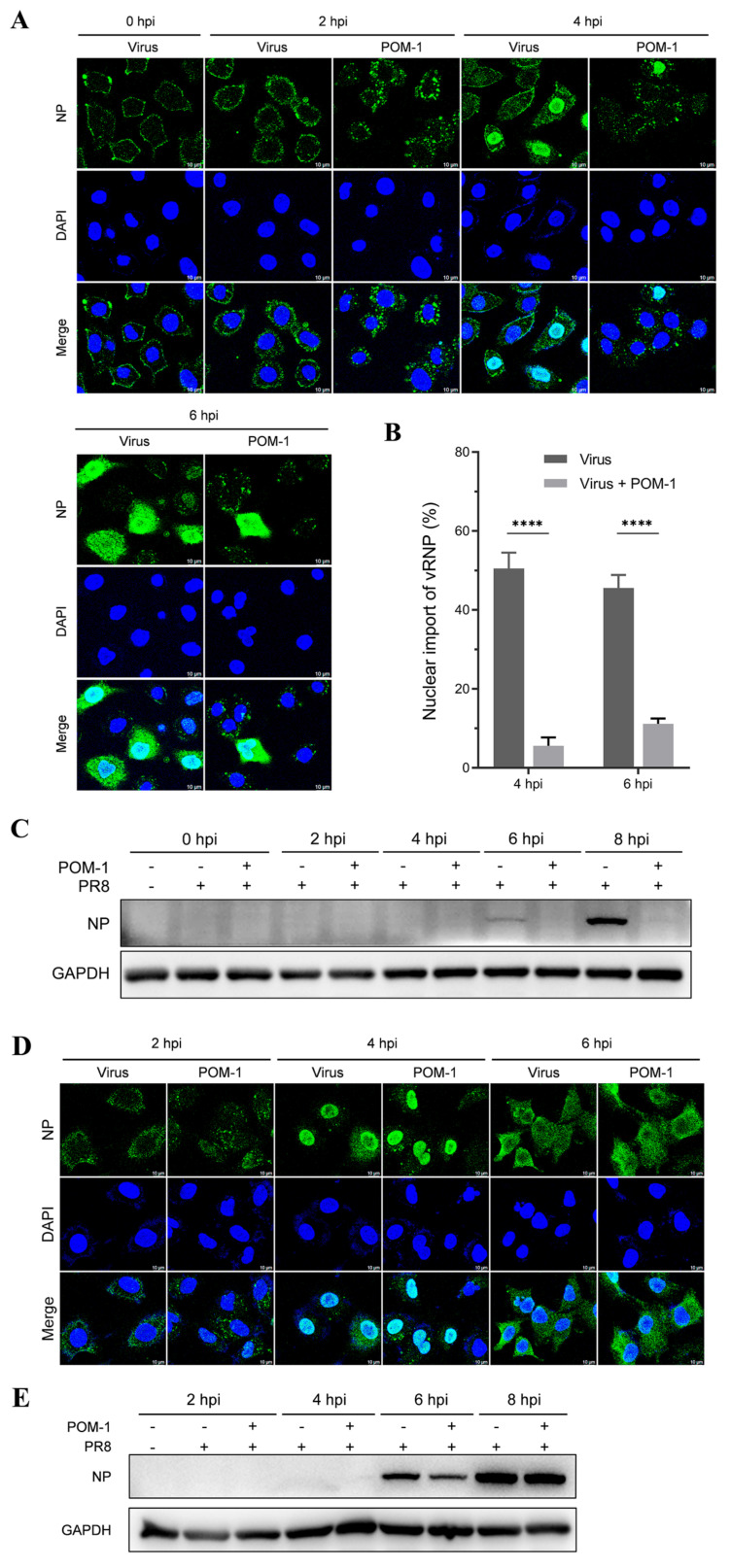
POM-1 causes virion aggregation in the cytoplasm and hinders the nuclear import of vRNP. (**A**) A549 cells were precooled at 4 °C for 30 min and infected with PR8 for 1 h. Subsequently, cells were washed, followed by the addition of medium containing POM-1 (100 µM) at 0 hpi and incubated at 37 °C. At different time points post-infection, infected cells (MOI = 5) were fixed and visualized using IFA staining for NP (green), while the nucleus was stained with DAPI (blue). (**B**) A549 cells were treated as described in (**A**) except for the used MOI of 1. Infected nuclei containing NP were considered imports of vRNP, while the percentage of cells with infected nuclei was calculated per field. The number of counted cells at 4 hpi for the virus control group and POM-1-treated group were 79 and 90, respectively, and for 6 hpi, they were 117 and 82. Values are expressed as the means ± SEM of three independent experiments. The statistical significance of the results is indicated by **** *p* < 0.0001. (**C**) A549 cells were treated as described in (**A**), except that lysed infected cells (MOI = 1) were subjected to Western blotting to determine NP protein levels. (**D**) A549 cells were precooled and infected with PR8 at 4 °C for 1 h. Then, the cells were washed twice with PBS and cultured at 37 °C in the presence of POM-1 (100 µM) for an additional hour; subsequently, they were washed again with PBS and cultured in a fresh DMEM medium. At different times post-infection, infected cells (MOI = 5) were fixed and visualized using IFA staining for NP protein expression (green), while the nucleus was stained with DAPI (blue). (**E**) A549 cells underwent treatment as described in (**D**), except that lysed infected cells (MOI = 1) were subjected to Western blotting analysis to determine NP protein levels.

Given that POM-1 induces virion aggregation during adsorption, we sought to investigate whether POM-1 retains its antiviral efficacy when administered after viral adsorption. Subsequently, a virus replication assay was performed by introducing POM-1 post-virus adsorption. Intriguingly, at 2 hpi, cytoplasmic virion aggregation was observed despite unaffected endocytosis upon late treatment with POM-1 compared to the untreated control group. At 4 and 6 hpi, the nuclear import of vRNP and NP expression were impeded by POM-1 treatment (Figure 5A). A quantitative analysis of vRNP nuclear import depicted in Figure 5B further substantiates the hindrance caused by POM-1 treatment. Additionally, Western blot analysis revealed the abrogation of NP expression following administration of POM-1 subsequent to virus adsorption (Figure 5C).

Our findings suggest that treatment with POM-1 after virus adsorption induces the cytoplasmic aggregation of virions and impedes the nuclear import of vRNP.

### 3.7. The Aggregation of Virions Caused by POM-1 in the Cytoplasm Is Reversible

Subsequently, we aimed to investigate the reversibility of aggregated virions in the cytoplasm. Influenza viruses were attached to A549 cells at 4 °C, followed by a PBS solution wash to remove unattached viruses. The cells were then cultured at 37 °C for 1 h with or without POM-1 and subsequently rewashed to remove POM-1, followed by continued culturing at 37 °C. Virus replication was assessed by examining NP expression and localization using IFA at different time points after virus infection. As illustrated in Figure 5D, at 2 hpi, one hour after the removal of POM-1, the cytoplasmic virion aggregation could still be observed; however, the size of virion aggregates gradually reduced over time and could not be seen at 6 hpi. Nuclear import of vRNP and NP export were observed, respectively, in POM-1-treated (during endocytosis) cells at 4 and 6 hpi, resembling those seen in untreated cells. These findings suggest that removing POM-1 results in reversible virion aggregation within the cytoplasm.

We conducted a virus replication experiment to validate the reversal of virion aggregation in the cytoplasm and restore the virus replication capacity upon POM-1 removal. We assessed viral NP expression through Western blot analysis. The results demonstrated that the elimination of POM-1 restored viral NP expression in infected cells to levels comparable with untreated cells (Figure 5E), indicating unaffected upstream events such as endocytosis and nuclear import of vRNP after POM-1 removal after virus endocytosis. Therefore, our findings suggested that POM-mediated virion aggregation after endocytosis is reversible upon POM-1 elimination.

### 3.8. POM-1 Has No Effect on the Release of Influenza Virions

**Figure 6:**
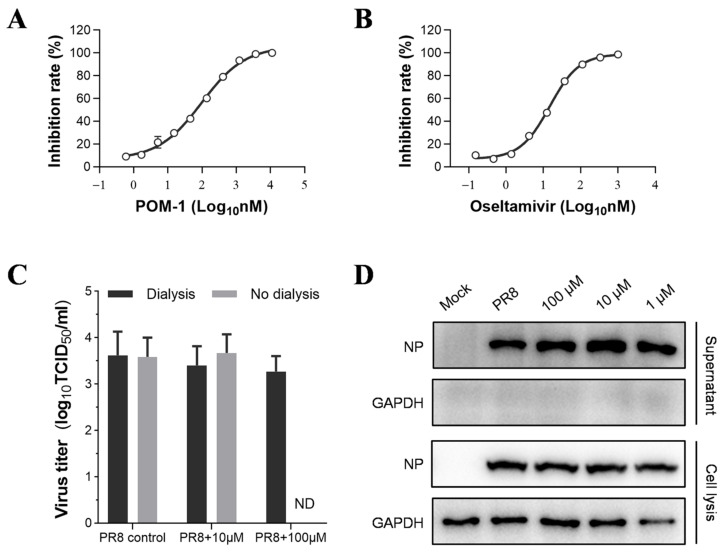
POM-1 does not impact the release of influenza virions. (**A**,**B**) In the neuraminidase inhibition assay, the PR8 virus was incubated with serially diluted POM-1 or oseltamivir at 37 °C for 1 h. Subsequently, the mixtures were incubated with MUNANA at 37 °C for 1 h, and the inhibition rate was calculated based on the fluorescence intensity measured using a microplate reader. The data are presented as means ± SEM from three independent experiments. (**C**) A549 cells were infected with PR8 at an MOI of 0.5 for 6 h, followed by the replacement of a culture medium with DMEM containing different concentrations of POM-1. At 10 hpi, supernatants were collected and assessed for infectious virion production using a TCID_50_ assay after dialysis to remove POM-1 or without dialysis. Data are presented as means ± SEM from three independent experiments. (**D**) Western blotting was performed to analyze viral NP levels in collected supernatants and total lysates in infected cells.

The surface antigens HA and NA are responsible for entering and releasing influenza viruses. We performed a hemagglutination inhibition assay to assess the impact of POM-1 on viral HA function. Our findings demonstrate that even at the highest concentration of 500 μM, POM-1 does not impede IAV-induced aggregation of erythrocytes. These results indicate that POM-1 does not influence erythrocyte agglutination mediated by viral HA. However, in a neuraminidase inhibition assay depicted in Figure 6A,B, we observed significant inhibition of neuraminidase activity by POM-1, which is similar to that of oseltamivir. The IC_50_ value for POM-1 was determined to be 97.76 nM, which is slightly higher than oseltamivir’s IC_50_ value of 14.76 nM. These results suggest that POM-1 has an inhibitory effect on the neuraminidase activity of the influenza virus.

To assess the impact of POM-1 on NA-mediated virion release, we evaluated virus replication through Western blot analysis and the production of progeny virions using the TCID_50_ assay. Following a six-hour viral infection period, cells were treated or left untreated with POM-1 for an additional 4 h. Notably, POM-1 treatment did not affect the expression of viral NP during the late stage of the virus life cycle (6–10 hpi) (Figure 6D). Moreover, this late-stage treatment with POM-1 had no discernible effect on the release of infectious virions (Figure 6C,D). These findings indicate that POM-1 does not inhibit virion release in the life cycle of influenza virus.

## 4. Discussion

The results of this study show that POM-1 has potent antiviral properties against influenza virus infection in vitro. POM-1 has a wide-range antiviral effect against H1N1 and H3N2 influenza A viruses, as well as a strain resistant to oseltamivir, on both A549 and MDCK cells. In the mechanism of action study, we initially demonstrated that POM-1 exerts its antiviral effect on influenza virus replication during the early stages of the viral life cycle, as determined by a time-of-addition assay. Subsequently, we investigated the impact of POM-1 treatment on virus adsorption, endocytosis, and nuclear import of vRNP during virus attachment and entry. Our findings revealed that POM-1 induces virion aggregation on the cell surface and in the cytoplasm, with stronger aggregation observed at high viral MOI and POM-1 concentrations. However, despite virion aggregation, overall, endocytosis remains unaffected. Significantly, the initiation of treatment during adsorption or virus entry does not compromise endocytosis efficiency. Conversely, nuclear import of vRNP is hindered by POM-1 treatment, leading to an impaired production of infectious influenza virions.

In addition, we observed that treatment with POM-1 induces the aggregation of influenza virus during adsorption or viral entry, and this aggregation is reversible upon drug removal. We hypothesize that the interaction between POM-1 and its viral or host targets exhibits non-specificity and reversibility. In a study conducted by Wall et al., it was also demonstrated that the inhibitory effect of POM-1 on cerebellar ATP breakdown can be readily reversed [30].

Furthermore, we found that removing POM-1 prior to the nuclear import of vRNP can restore virus replication capacity, albeit with a slight delay. Notably, it may enhance virus replication under high viral infection and concentrated short-term POM-1 treatment. Therefore, for effective influenza virus infection treatment, maintaining POM-1 at an optimal concentration beyond a few cycles of virus replication is crucial to prevent the potential promotion of viral replication.

As POM-1 falls under the category of polyoxometalates, they consist of clusters comprising early-transition metal cations bridged by oxide anions. In our literature search for polyoxometalates exhibiting antiviral activity against influenza viruses in terms of efficacy and mechanisms of action, we found that most polyoxometalates demonstrated similar efficacy but varied mechanisms of action. For instance, PM-504 and PM-523 exhibited EC_50_ values of 1.3 and 2.4 μM against IAV, respectively, with corresponding selectivity indices (SIs) exceeding 300 and 167 [27]. While PM-504 inhibited the binding between IAV and cells, PM-523 did not; however, it was identified to inhibit fusion between the viral envelope and cell membrane. HS-058 displayed inhibitory effects against influenza viruses A and B with EC_50_ values of 1.4 and 21.8 μM, respectively [31]. When HS-058 was added to the culture either 1 h before viral adsorption followed by removal or after virus adsorption followed by removal after treatment for 1.5 h, it did not exhibit inhibitory effects on IAV replication. The compound showed consistent protective effects when cells were exposed to POM-4960 before, during, or after IAV infection [32].

Our study, and reports from other researchers, suggest that polyoxometalates exert their effects during the early stages of influenza virus replication. In vitro experiments have demonstrated that a series of germanium- or silicon-centered heteropolytungstates (polyoxometalates) with Barrel, Keggin, or double-Keggin structures exhibit the highest efficacy when administered at the onset of viral replication [19]. Furthermore, intraperitoneal treatment with five highly active polyoxometalates was effective against influenza B virus infections in mice when initiated 4 h before virus exposure. However, initiating therapy 8 h after virus exposure was ineffective in the in vivo infection.

POMs typically possess negative charges attributed to their structural characteristics, enabling them to engage in electrostatic interactions and hydrogen bonding with host factors [33,34]. Overexpression of protein kinase CK2 leads to abnormally elevated activity associated with various diseases such as cancer, diabetes, and COVID-19. The inhibitory potential of POMs against CK2 activity exhibited a rough correlation with the size and charge in the POM; larger and highly charged POMs generally displayed greater efficacy due to stronger binding interactions [35]. Ru_4_POM, a potent inhibitor targeting Ser/Thr protein kinase CK2, binds to the positively charged substrate binding region of the enzyme through electrostatic interactions. This interaction triggers enzyme dimerization, leading to subsequent inactivation [36].

The Keggin-type POMs were discovered to effectively inhibit tyrosinase activity by competitively binding to the enzyme, primarily through increased interactions with Cu^2+^ ions. Additionally, the amino acid residue can form van der Waals, cation-π, and hydrogen bonds, forming a reversible non-covalent complex [37]. For instance, POM-1 binds to specific sites on the target molecule, leading to the functional modulation or inhibition of its activity.

POM-1 is known as an inhibitor of Ecto-NTPDases that inhibits NTPDase 1/2/3 [30,38]. Since it inhibits ATP breakdown, we asked whether its antiviral activity is related to increased ATP levels in the cell culture. To test this hypothesis, we infected A549 cells with the PR8 influenza virus in the presence of increasing concentrations of ATP. We found that the viral replication was not affected by ATP at up to 100 μM, suggesting that the antiviral effect of POM-1 is an off-target effect.

In addition to the current findings presented in this study, further research is required to explore the potential of POM-1 as an anti-influenza drug. It is crucial to establish a more precise mechanism of action to determine which stage of entry is obstructed and which target POM-1 interacts with. Moreover, in vivo testing should be conducted to assess the antiviral efficacy of POM-1. Additionally, it is also necessary to investigate whether POM-1 regulates influenza-induced inflammation in the lungs of infected animals. However, the unique mechanism of action and broad range of effectiveness make POM-1 a promising therapeutic candidate for treating influenza, and further investigation is warranted.

## Data Availability

The raw data supporting the conclusions of this article will be made available by the authors on request.
